# Unifying Serum Creatinine and Urine Output in a Single On-Time AKI Severity Criterion: Is It All About the Rate of Creatinine Being Excreted by the Kidneys?

**DOI:** 10.3390/diagnostics16020181

**Published:** 2026-01-06

**Authors:** Alexandre Toledo Maciel

**Affiliations:** Imed Group, Research Department, Adult ICU, Hospital São Camilo Pompéia, São Paulo 05022-001, Brazil; alexandre.toledo@imedgroup.com.br

**Keywords:** acute kidney injury, KDIGO criteria, excreted mass of creatinine, urine output, serum creatinine, urinary creatinine excretion, prognosis

## Abstract

Serum creatinine (sCr) and urine output (UO) have long been considered the cornerstones of acute kidney injury (AKI) severity criteria. Many articles were previously published discussing the prognostic relevance of fulfilling either one or both AKI criteria. However, sCr and UO must not be considered independent variables because they are physiologically linked despite having distinct chronologies as AKI markers. An increase in sCr is a late manifestation of decreased renal function and body creatinine accumulation and not an on-time surrogate for a decreasing glomerular filtration rate. On the other hand, oliguria is not a single entity, and its interpretation relies on urine’s biochemical composition as well as its threshold pathological output value, which is somewhat controversial. In the present article, the current practice of evaluating sCr and UO separately is questioned and the idea that they can eventually be considered different expressions of the same variable of interest (the urine creatinine excretion) is highlighted.

## 1. Introduction

Acute kidney injury (AKI) is a threatened complication of critical illness with a high morbimortality, directly related to the duration and severity of renal function impairment [1]. To quantify such impairment, many AKI stage criteria were developed using increments in serum creatinine (sCr) and decrements in urine output (UO) as markers of decreased renal function. Currently, the most commonly used are the Kidney Disease Improving Global Outcomes (KDIGO) criteria [2] which, similar to previous Acute Kidney Injury Network (AKIN) [3] and Risk, Injury, Failure, Loss, End-stage (RIFLE) [4] criteria, are based on absolute or percentage increases in sCr within a certain timeframe (2 or 7 days, respectively) or a reduced UO evaluated in a shorter timeframe (6, 12 or 24 h).

The limitations and drawbacks of sCr and UO as markers of renal function have been well described previously by many authors [5,6]. Increases in sCr are usually the preferred criterion because sCr is easily assessed and measured. Conversely, UO is considered to be of more complex measurement and interpretation, having many variables that can affect its value. Adding UO to sCr-based AKI criteria usually increases the incidence of AKI [7,8] due to its augmented sensitivity, as decreases in UO frequently precede increases in sCr and only a percentage of oliguric patients will actually have increments in sCr. However, the cutoff UO value that can truly be considered kidney dysfunction is still controversial and some studies have suggested a lower threshold than the classic 0.5 mL/kg/h [9,10]. UO tends to be a more dynamic and on-time parameter than sCr because it is usually continuously measured while sCr is measured punctually and its increment is a result of a previous significantly impaired urinary creatinine excretion (CrUexc). Therefore, sCr is not a surrogate for on-time renal impairment and this is the main reason why it is a late and less versatile AKI marker.

## 2. Considerations About sCr and UO as Renal Function Surrogates

### 2.1. Are Both Increased sCr and Decreased UO Only Markers of Impaired Creatinine Excretion?

Increases in sCr due to decreases in glomerular filtration rate (GFR) are preceded by impaired CrUexc, no longer counterbalancing creatinine production, leading to body creatinine accumulation. Considering that increases in sCr are just an epiphenomenon of a reduced CrUexc, which ultimately defines AKI severity, the role of UO as a prognostic marker would also be related, at least in part, to a reduction in CrUexc. It is important to highlight that UO and CrUexc are not linearly correlated because there is a “hidden”, frequently not assessed variable, which is the urine creatinine concentration ([CrU]).

### 2.2. Can Urine Creatinine Concentration Stratify Oliguria-Related Prognosis in Critically Ill Patients?

It is, therefore, noteworthy that the kidneys are able to change the [CrU] so that, for the same UO, the CrUexc may vary widely [11]. Moreover, there is a delay between decreases in UO and increases in sCr, depending on several variables, including current creatinine production by muscles, the magnitude of the UO decrease, the [CrU] and the body creatinine volume of distribution [12]. In this context, the same UO (in mL/kg/h) may have different implications and interpretations but the concept of “quality”, not only “quantity” of urine, is currently undervalued by most Intensive Care Unit (ICU) practitioners and not properly explored in the medical literature.

### 2.3. Urine Creatinine Concentration: A Key Element in the Evaluation of Oliguria and the Subsequent Behavior of sCr

**Figure 1:**
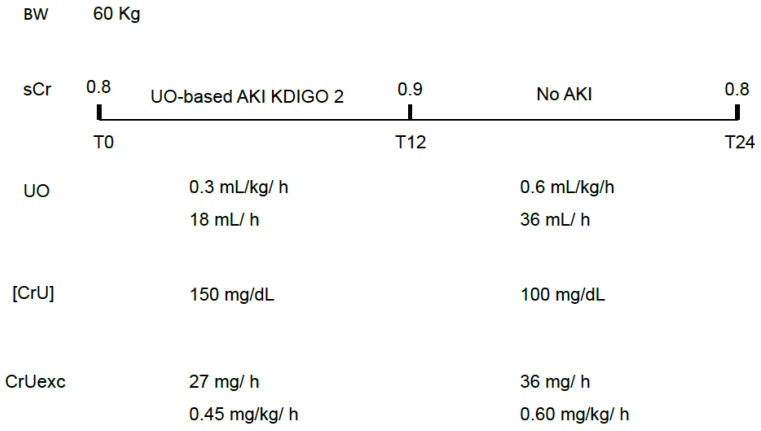
A hypothetical situation in which transient oliguria leads to UO-based AKI criteria not followed by increases in sCr. In this case, only mild and transient decreases in CrUexc have occurred. An increase in UO is not mandatorily followed by a proportional decrease in [CrU] and this is why the excreted mass of creatinine has improved, precluding further increases in sCr. sCr: serum creatinine; UO: urine output; [CrU]: urine creatinine concentration; AKI: acute kidney injury; KDIGO: Kidney Disease Improving Global Outcomes; CrUexc: urine creatinine excretion as mg of creatinine per kilogram per hour, T12: 12 h after initial assessment (T0); T24: 24 h after initial assessment (T0); BW: body weight.

**Figure 2:**
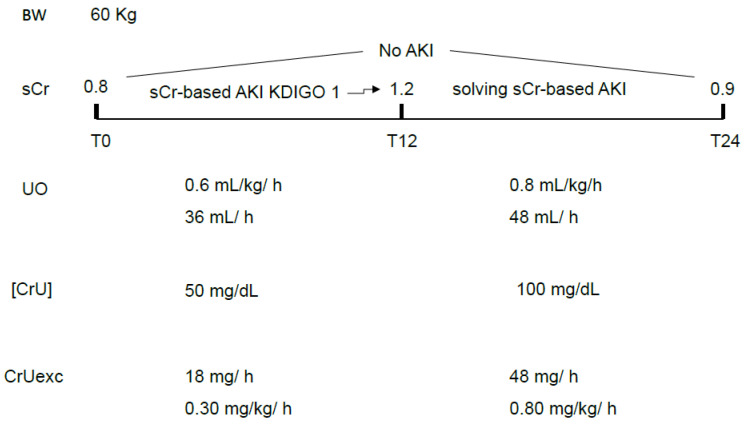
A hypothetical situation in which a very transient sCr-based AKI would not be diagnosed except if sCr was also measured 12 h and not only 24 h after initial measurement (T0). UO was within normal values throughout the 24 h period but, in the first half of the day, the CrUexc was compromised, recovering in the second half. It is noteworthy that, in this example, variations in the [CrU] were responsible for the CrUexc decrement and subsequent recovery, leading to a rapid increase and decrease in sCr. A transient oliguria might also contribute and exacerbate this movement in CrUexc. sCr: serum creatinine; UO: urine output; [CrU]: urine creatinine concentration; AKI: acute kidney injury; KDIGO: Kidney Disease Improving Global Outcomes; CrUexc: urine creatinine excretion as mg of creatinine per kilogram per hour, T12: 12 h after initial assessment (T0); T24: 24 h after initial assessment (T0); BW: body weight.

Without knowing the [CrU], it is hard to predict the effect of decreases in UO on the sCr and how much time will be required for changes in the former to reverberate in the latter. Some studies have suggested that decreases in UO carry a poor prognosis independently of increases in sCr [13,14,15,16]. Patients fulfilling only UO-based criteria for AKI (no increases in sCr) would have a worse outcome than patients with no AKI [17]. At this point, some possibilities must be considered:

(1) The independent prognostic relevance of oliguria is directly and solely related to decreases in CrUexc.
  Decreases in CrUexc due to reduced UO but not affecting sCr raise two possibilities:
(1.1) Oliguria may be transient, compromising CrUexc and leading to UO-based AKI diagnosis but not being severe and long enough to allow sCr to increase 0.3 mg/dL in 2 days or 50% in 7 days (transient and short-lasting decreases in CrUexc). In this case, the magnitude and/or duration of the CrUexc decrease are expected to be lower than that which leads to a rise in sCr (Figure 1).

(1.2) UO-based criteria are measured in hours, and sCr-based criteria are measured in days. Depending on the duration of the observation period and severity of CrUexc decrement, it is possible that the observation period was not long enough to diagnose significant increases in sCr compatible with AKI (long-lasting decreased CrUexc but short observation period). Conversely, having most of the intensivists the practice of measuring sCr only once a day also leads to undiagnosed, very transient sCr-based AKI [18], which is characterized by increases and subsequent decreases in sCr greater than 0.3 mg/dL over 24 h (Figure 2). This situation is particularly common postoperatively, with an abrupt decrease in CrUexc (whether with or without simultaneous oliguria) being of sufficient magnitude to significantly increase sCr (rapid body creatinine accumulation) but lasting only a few hours.

**Figure 3:**
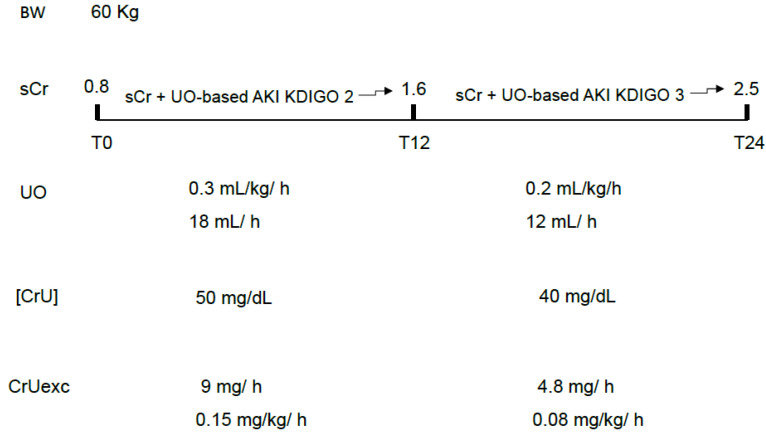
An example of the most severe and fast decrement in CrUexc due to both decreases in UO and [CrU]. In this case, increases in sCr tend to be more rapid and of greater magnitude, likely serving as a surrogate for the most abrupt loss of GFR and body creatinine accumulation. sCr: serum creatinine; UO: urine output; [CrU]: urine creatinine concentration; AKI: acute kidney injury; KDIGO: Kidney Disease Improving Global Outcomes; CrUexc: urine creatinine excretion as mg of creatinine per kilogram per hour, T12: 12 h after initial assessment (T0); T24: 24 h after initial assessment (T0); BW: body weight.

In both cases, the prognostic relevance of oliguria would be due to an initial and significant decrease in CrUexc, which could be intense but short-lasting (no significant or very transient increases in sCr) or less severe but long-lasting, leading to increases in sCr only after the observation period. In other words, even a decrease in CrUexc not severe enough to be accompanied by simultaneous or sustained increases in sCr may have prognostic relevance. The worst prognosis usually attributed to the simultaneous presence of UO and sCr AKI-based criteria might be merely a surrogate for a more intense decrease in CrUexc (Figure 3).

(2) The independent prognostic relevance of oliguria is not only related to decreases in CrUexc.
  If this is the case, the prognostic role of oliguria must be divided in

(2.1) with simultaneous decrement in CrUexc

Studies would be needed showing that oliguria carries a poor prognosis independently of the simultaneous decrease in CrUexc.

(2.2) with no simultaneous decrement in CrUexc

Studies would be needed showing that oliguria with no decrements in CrUexc (due to high [CrU]) also carries a worse prognosis than patients with no UO-based AKI criteria.

### 2.4. Can the Estimated (or Measured) Creatinine Clearance Solve This Prognostic Issue by Combining UO and sCr?

Decreases in GFR can be estimated by calculating the creatinine clearance. Many formulas were proposed but the Modification of Diet in Renal Disease (MDRD) formula [19] was the one chosen by the KDIGO AKI criteria. However, creatinine clearance calculation is of limited interpretation in AKI since a single and static value of sCr is used in the formula in a situation of actively declining GFR. Both estimated and, particularly, measured creatinine clearance are only ideal for a stable renal function. An increased sCr will inevitably reduce both calculated and measured creatinine clearance, although sCr value is a consequence of previous GFR, not a surrogate for the current GFR.

### 2.5. Excreted Mass of Creatinine per Hour: An Alternative to Evaluate Changes in GFR Without the Need of sCr Assessment?

The excreted mass of creatinine per hour is a parameter that excludes sCr from the on-time evaluation of renal function. It is reasonable to think that, if sCr is a consequence of previous impaired renal function and, therefore, not a measure of current renal function itself, its inclusion will disturb the on-time GFR assessment except if GFR is stable during the entire observation period. The advantage of measuring the excreted mass of creatinine is that it includes both UO and [CrU] in its calculation. [CrU] assessment is very helpful because it is probably the most relevant point of connection between UO and sCr [11]. If acute decreases in CrUexc are the ultimate cause of poor prognosis, reflecting impaired GFR, a decreasing excreted mass of creatinine will be evident, in the presence or not of oliguria, and preceding increases in sCr. Moreover, it is well established that a low baseline CrUexc increases mortality as a surrogate for a decreased muscle mass in critically ill patients [20,21]. Independently of age and gender, CrUexc is expected to decrease 1–2% a day, attributed to daily loss of muscle mass [20,22]. While monitoring renal function, decreases in CrUexc are expected to be significantly more than just 1–2% a day in AKI patients. Hence, abrupt decreases in the excreted mass of creatinine are supposed to be related to GFR, not to muscle loss or reduced creatine metabolism, producing less creatinine. Conversely, significant and persistent decreases in CrUexc not followed by increases in sCr may be alternatively interpreted as simultaneous reduced muscular creatinine production rate [23] considering a stable systemic creatinine volume of distribution.

## 3. Relevant Limitations of the Excreted Mass of Creatinine as a Renal Function Marker

**Figure 4:**
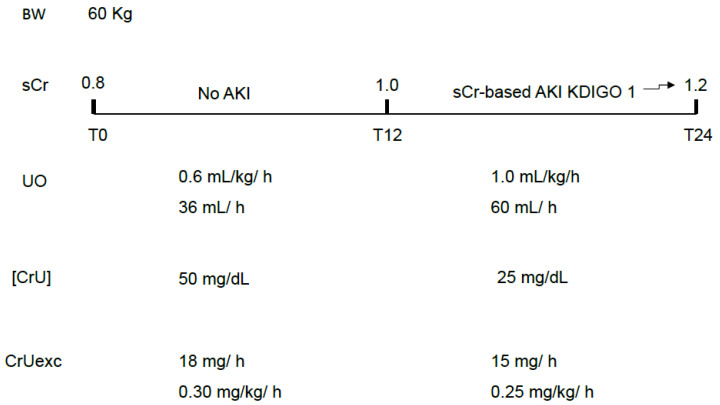
Non-oliguric AKI as a classic example of AKI that is basically attributed to a decreased [CrU]. The theoretically “unexpected” gradual increase in sCr over 24 h would not be a surprise if CrUexc was being monitored instead of UO only. sCr: serum creatinine; UO: urine output; [CrU]: urine creatinine concentration; AKI: acute kidney injury; KDIGO: Kidney Disease Improving Global Outcomes; CrUexc: urine creatinine excretion as mg of creatinine per kilogram per hour, T12: 12 h after initial assessment (T0); T24: 24 h after initial assessment (T0); BW: body weight.

In order to prevent body creatinine accumulation, which is indirectly diagnosed by increases in sCr, the excreted mass of creatinine must equal the produced mass of creatinine at the same time interval. The first limitation of the excreted mass of creatinine is not knowing, in clinical practice, the creatinine production rate. A value around 0.5–1 mg/kg/h is usually considered physiological but critical illness usually modifies this rate and the patient’s weight is not a confident surrogate for his muscle mass. Besides that, age and gender also interfere with this estimation. However, a sequential decrease in the excreted mass of creatinine can be a sign of progressive GFR impairment, even in the absence of simultaneous decreasing UO, as occurs with diuretic use. It also facilitates and anticipates the non-oliguric AKI diagnosis (Figure 4). As previously mentioned, it is important to highlight that the excreted mass of creatinine is related not only to GFR but also to body muscle mass, although abrupt decreases in the excreted mass (in hours) are expected to be mainly related to GFR impairment.

Another limitation to its use is the need of an indwelling urinary catheter for measuring the UO precisely in a certain timeframe. As a possible surrogate for using the excreted mass of creatinine, the KU/CrU ratio has been proposed and can be measured in a spot urine sample [24]. A high KU/CrU ratio (>0.6) usually corresponds to a low excreted mass of creatinine per hour, but additional studies are needed to confirm the correlation between these variables.

Finally, decreases in the CrUexc can be mitigated by tubular creatinine secretion so that decreases in GFR are usually greater and earlier than decreases in the excreted mass of creatinine. The magnitude and prognostic relevance of this delay are currently unknown.

## 4. Conclusions

On-time monitoring of renal function is a challenge for critical care practitioners and oliguria is not a single entity, having different meanings and possibly different outcomes depending on its biochemical composition. [CrU] assessment is an undervalued parameter that may facilitate oliguria interpretation as well as anticipate the diagnosis of a declining GFR that may occur in the absence of significant decreases in the UO. Future studies should include [CrU] as part of sequential CrUexc assessment, believing that, in the short term, significant decrements in the excreted mass of creatinine are mainly due to impaired GFR and not due to decrements in the creatinine production by muscles. The usefulness of measuring [CrU] and the excreted mass of creatinine is that they may abolish the separated assessment of sCr and UO, as currently preconized by KDIGO-based AKI criteria, since both are probably included in the same spectrum of disease severity quantified by CrUexc as a single (and simple) parameter. The relevance and clinical impact of CrUexc monitoring in critically ill patients should be tested in large, multicenter trials to better define whether or not it improves and anticipates our perception of both renal function impairment and recovery.

## Data Availability

Not applicable.

## References

[1] Pickkers P., Darmon M., Hoste E., Joannidis M., Legrand M., Ostermann M., Prowle J.R., Schneider A., Schetz M. (2021). Acute kidney injury in the critically ill: An updated review on pathophysiology and management. Intensive Care Med..

[2] Kellum J.A., Lameire N., Aspelin P., Barsoum R.S., Burdmann E.A., Goldstein S.L., Herzog C.A., Joannidis M., Kribben A., Levey A.S. (2012). Kidney disease: Improving global outcomes (KDIGO) acute kidney injury work group. KDIGO clinical practice guideline for acute kidney injury. Kidney Int. Suppl..

[3] Mehta R.L., Kellum J.A., Shah S.V., Molitoris B.A., Ronco C., Warnock D.G., Levin A. (2007). Acute Kidney Injury Network. Acute Kidney Injury Network: Report of an initiative to improve outcomes in acute kidney injury. Crit. Care.

[4] Venkataraman R., Kellum J.A. (2007). Defining acute renal failure: The RIFLE criteria. J. Intensive Care Med..

[5] Ostermann M., Legrand M., Meersch M., Srisawat N., Zarbock A., Kellum J.A. (2024). Biomarkers in acute kidney injury. Ann. Intensive Care.

[6] Wen Y., Parikh C.R. (2021). Current concepts and advances in biomarkers of acute kidney injury. Crit. Rev. Clin. Lab. Sci..

[7] Quan S., Pannu N., Wilson T., Ball C., Tan Z., Tonelli M., Hemmelgarn B.R., Dixon E., James M.T. (2016). Prognostic implications of adding urine output to serum creatinine measurements for staging of acute kidney injury after major surgery: A cohort study. Nephrol. Dial. Transplant..

[8] Wu L., Li Y., Zhang X., Chen X., Li D., Nie S., Li X., Bellou A. (2023). Prediction differences and implications of acute kidney injury with and without urine output criteria in adult critically ill patients. Nephrol. Dial. Transplant..

[9] Machado G.D., Santos L.L., Libório A.B. (2024). Redefining urine output thresholds for acute kidney injury criteria in critically Ill patients: A derivation and validation study. Crit. Care.

[10] Mizota T., Yamamoto Y., Hamada M., Matsukawa S., Shimizu S., Kai S. (2017). Intraoperative oliguria predicts acute kidney injury after major abdominal surgery. Br. J. Anaesth..

[11] Maciel A.T. (2016). Back to Basics: Is There a Good Reason to Not Systematically Measure Urine Creatinine in Acute Kidney Injury Monitoring?. Nephron.

[12] Maciel A.T. (2025). Giving urine biochemistry a second chance in acute kidney injury monitoring. World J. Crit. Care Med..

[13] Malbrain M.L.N.G., Tantakoun K., Zara A.T., Ferko N.C., Kelly T., Dabrowski W. (2024). Urine output is an early and strong predictor of acute kidney injury and associated mortality: A systematic literature review of 50 clinical studies. Ann. Intensive Care.

[14] Macedo E. (2016). Urine output assessment as a clinical quality measure. Nephron.

[15] Vaara S.T., Parviainen I., Pettilä V., Nisula S., Inkinen O., Uusaro A., Laru-Sompa R., Pulkkinen A., Saarelainen M., Reilama M. (2016). Association of oliguria with the development of acute kidney injury in the critically ill. Kidney Int..

[16] Bianchi N.A., Stavart L.L., Altarelli M., Kelevina T., Faouzi M., Schneider A.G. (2021). Association of Oliguria with Acute Kidney Injury Diagnosis, Severity Assessment, and Mortality among Patients with Critical Illness. JAMA Netw. Open.

[17] Macedo E., Malhotra R., Bouchard J., Wynn S.K., Mehta R.L. (2011). Oliguria is an early predictor of higher mortality in critically ill patients. Kidney Int..

[18] Maciel A.T., Nassar A.P., Vitorio D. (2016). Very Transient Cases of Acute Kidney Injury in the Early Postoperative Period after Cardiac Surgery: The Relevance of More Frequent Serum Creatinine Assessment and Concomitant Urinary Biochemistry Evaluation. J. Cardiothorac. Vasc. Anesth..

[19] Levey A.S., Bosch J.P., Lewis J.B., Greene T., Rogers N., Roth D. (1999). A more accurate method to estimate glomerular filtration rate from serum creatinine: A new prediction equation. Modification of Diet in Renal Disease Study Group. Ann. Intern. Med..

[20] Hessels L., Koopmans N., Gomes Neto A.W., Volbeda M., Koeze J., Lansink-Hartgring A.O., Bakker S.J., Straaten H.M.O.-V., Nijsten M.W. (2018). Urinary creatinine excretion is related to short-term and long-term mortality in critically ill patients. Intensive Care Med..

[21] Hsu C.Y., Wu Y.L., Cheng C.Y., Lee J.D., Huang Y.C., Lee M.H., Wu C.Y., Hsu H.L., Lin Y.H., Huang Y.C. (2015). Low Baseline Urine Creatinine Excretion Rate Predicts Poor Outcomes among Critically Ill Acute Stroke Patients. Curr. Neurovasc. Res..

[22] Fazzini B., Märkl T., Costas C., Blobner M., Schaller S.J., Prowle J., Puthucheary Z., Wackerhage H. (2023). The rate and assessment of muscle wasting during critical illness: A systematic review and meta-analysis. Crit. Care.

[23] Yamamoto N., Tojo K., Mihara T., Maeda R., Sugiura Y., Goto T. (2025). Creatinine production rate is an integrative indicator to monitor muscle status in critically ill patients. Crit. Care.

[24] Maciel A.T. (2025). Introducing the “urine biochemical approach”: An alternative tool for improving acute kidney injury monitoring in critically ill patients. Front. Nephrol..

